# Among Soldiers' Children

**Published:** 1889-06-29

**Authors:** 


### Among Soldiers' Children.

The Duchess of Albany visited Hampstead on the 18th, to distribute prizes to the pupils of the Soldiers' Daughters' School, an institution which was founded thirty-four years ago, in great part through the exertions of the late General Boileau, and has during that time trained 1,041 girls for domestic service or teaching. The school does not let the girls go wholly out of sight when their education is finished; they can come back when out of work, and find a home there. At present, there are 164 girls in the school, and during the last year there has been only one death among them (from rapid consumption), and no other serious illness. It is to be regretted that there are between twenty and thirty beds vacant for a lack of funds, though the school is on the whole well supported in military circles, both by individuals and regiments.

The ceremony took place in a marquee in the pleasant and spacious garden behind the school, in front of which the girls were ranged. They presented quite a military appearance in their red frocks and hats trimmed with royal blue; and they had enough of manly spirit to welcome Her Royal Highness with three ringing treble cheers. Very well trained are they, too, for they received their prizes with a deep courtesy, and backed out of the royal presence as gracefully as any lady could do at Buckingham Palace. Two very touching points there were in the day's proceedings. One was the singing of the "Soldier's

Daughter's Song," which is set to the pathetic tune of "Auld Lang Syne," and begins:

"Victoria, Victoria, we hail thy gentle rule,
Victoria, the Patroness of the Soldiers' Daughters' School."

The other was when four girls presented the Duchess with a pretty work-basket, in which lay two dolls dressed by the pupils for her children. Throughout the whole of the ceremony Her Royal Highness's interest in the proceedings had been evident and genuine; but it was clear that this gift touched her deeply. A flush of pleasure came into her kindly face as she thanked the little donors, and she examined the dainty little garments with the loving touch of a connoisseur. Among the company present were Lord Napier of Magdala, the President of the school, Major-General Ravenhill, Colonel Bowness-Fischer, and many other distinguished officers. Major-General Ravenhill, as chairman of the committee, spoke of the admirable discipline of the home, due to the care and energy of the matron, Miss K. Bartlett, but confessed that he was distressed at one thing. He wanted a cot at some convalescent home, to which they could send delicate pupils in turn. But before the proceedings ended he was able to announce that a lady (Miss Breton) had promised to provide the desired cot. "This," said the General, "has made me a happy man." At the request of Lord Napier, the Duchess of Albany has consented to become a patroness of the school.

